# Effect of microgravity on mechanical loadings in lumbar spine at various postures: a numerical study

**DOI:** 10.1038/s41526-023-00253-8

**Published:** 2023-02-15

**Authors:** Biao Wu, Xin Gao, Bing Qin, Michele Baldoni, Lu Zhou, Zhiyu Qian, Qiaoqiao Zhu

**Affiliations:** 1 Department of Biomedical Engineering, Nanjing University of Aeronautics and Astronautics, Nanjing, China; 2 Department of Biomedical Engineering, University of Miami, Miami, FL USA

**Keywords:** Orthopaedics, Biomedical engineering

## Abstract

The aim of this study was to quantitatively analyze the mechanical change of spinal segments (disc, muscle, and ligament) at various postures under microgravity using a full-body musculoskeletal modeling approach. Specifically, in the lumbar spine, the vertebra were modeled as rigid bodies, the intervertebral discs were modeled as 6-degree-of-freedom joints with linear force-deformation relationships, the disc swelling pressure was deformation dependent, the ligaments were modeled as piecewise linear elastic materials, the muscle strength was dependent on its functional cross-sectional area. The neutral posture and the “fetal tuck” posture in microgravity (short as “Neutral 0G” and “Fetal Tuck 0G”, in our simulation, the G constant was set to 0 for simulating microgravity), and for comparison, the relaxed standing posture in 1G and 0G gravity (short as “Neutral 1G” and “Standing 0G”) were simulated. Compared to values at Neutral 1G, the mechanical response in the lower spine changed significantly at Neutral 0G. For example, the compressive forces on lumbar discs decreased 62–70%, the muscle forces decreased 55.7–92.9%, while disc water content increased 7.0–10.2%, disc height increased 2.1–3.0%, disc volume increased 6.4–9.3%, and ligament forces increased 59.5–271.3% at Neutral 0G. The fetal tuck 0G reversed these changes at Neutral 0G back toward values at Neutral 1G, with magnitudes much larger than those at Neutral 1G. Our results suggest that microgravity has significant influences on spinal biomechanics, alteration of which may increase the risks of disc herniation and degeneration, muscle atrophy, and/or ligament failure.

## Introduction

Microgravity exposure causes higher rates of back pain and disc herniations in astronauts^1,2^. Studies show that 52% of astronauts report spinal pain during their space mission, with 86% of which occurred in the lower back^1^. The incidence of intervertebral disc herniation in astronauts returned back to the earth from microgravity is much higher compared to that of matched control on the earth, it is 4.3 times higher for lumbar discs, with the highest risk appeared in the first year after return to the earth^3^, and 21.4 times higher for cervical discs^2^. The reason for much higher risks of low back pain and disc herniation in microgravity is not clear yet, some researchers proposed that intervertebral disc swelling due to unloading in microgravity may be a possible mechanism^2^. Thornton et al. showed that the stature increased around 4–6 cm (3% of stature) in microgravity^4^. Recently Young and Rajulu reported that seated height increased by 4% on average in an in-flight study^5^. The height increase in microgravity was thought mainly caused by spinal elongation^6^ through disc swelling and spinal curvature change^5^. Intervertebral disc swelling in microgravity has not been measured directly, though. However, it is reported that body height changes diurnally following the circadian rhythm on the earth, that is, a person is about 1.1% taller in the morning than at night^7^, due to that intervertebral disc imbibes/extrudes water during the unloading at night/loading at day, causing the intervertebral disc height to fluctuate diurnally. How much does human disc swell under microgravity are largely unknown yet, and how these swelling changes in the discs affect adjacent spinal segments mechanically are also largely unknown yet.

**Fig. 1:**
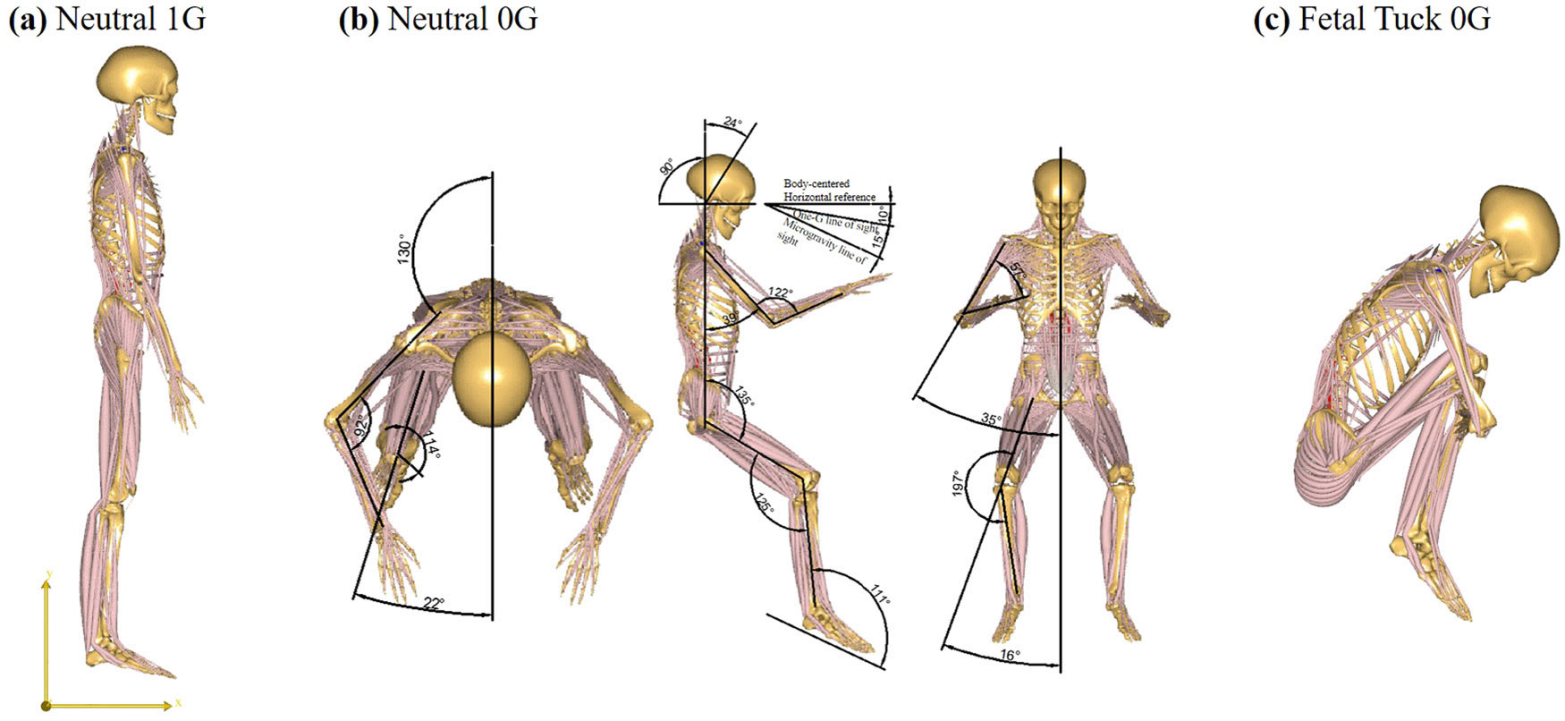
The postures simulated in 1G gravity and microgravity. **a** Relaxed standing posture in 1G gravity (Neutral 1G); this posture was also used in microgravity for comparison (Standing 0G), **b** relaxed floating posture in microgravity (Neutral 0G), and **c** fetal tuck posture in microgravity (Fetal Tuck 0G).

In microgravity, the neutral body posture (relaxed floating) was found quite different from the neutral posture (relaxed standing) in a gravitational environment, in which the torso was semi-crouched, arms and legs flexed, head and neck bent forward^8–10^ (Fig. 1). How are the spinal segments loaded mechanically under this posture in microgravity are unknown, whether the mechanical loadings among the spinal segments were different in microgravity from those at neutral standing in 1G gravity, and whether these difference (if any) possibly relate to lower back pain and/or disc herniation are also largely unknown. In addition, some astronauts claimed that low back pain is relieved by periodic “fetal tuck posture” in microgravity, that is, curling knees to the chest posture^11^ (Fig. 1). How does this “fetal tuck” posture relieve lower back pain biomechanically and whether this posture is mechanically safe to spinal health are also largely unknown, yet of great interests to us.

Thus, the aim of this study was to quantitatively analyze the mechanical change of various spinal segments in the lower back, including disc load, disc swelling, disc morphology (height, cross-sectional area, volume), muscle forces, and ligament forces at neutral and “fetal tuck” postures under microgravity using a musculoskeletal modeling approach. This study is important for understanding the biomechanical mechanisms of microgravity-related lower back pain and disc herniations, and this computational model is helpful in guiding future design and development of spinal countermeasures under microgravity.

## Results

**Fig. 2:**
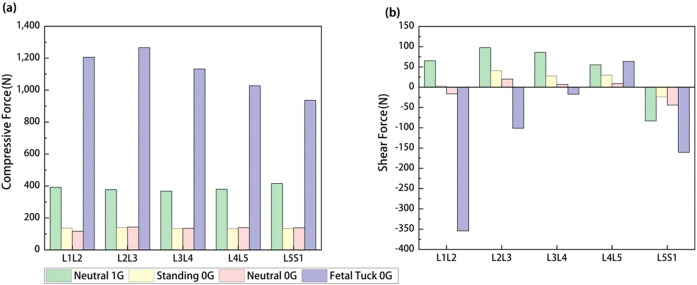
Comparison of mechanical forces on lumbar discs among various postures in 1G gravity and microgravity. **a** Compressive force and **b** shear force among Neutral 1G, Standing 0G, Neutral 0G, and Fetal Tuck 0G.

**Fig. 3:**
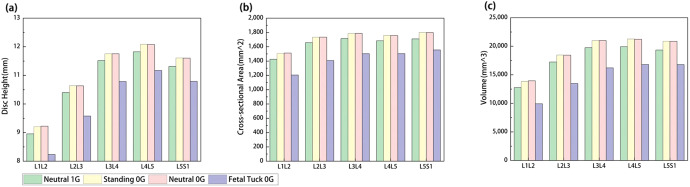
Comparison of disc morphology change among various postures in 1G gravity and microgravity. **a** Disc height, **b** cross-sectional area, and **c** disc volume change among Neutral 1G, Standing 0G, Neutral 0G, and Fetal Tuck 0G.

**Fig. 4:**
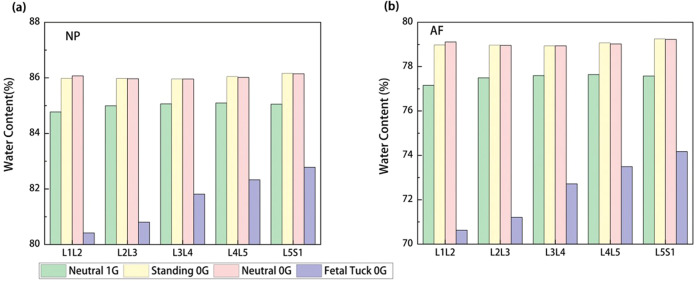
Comparison of water content in lumbar discs among various postures in 1G gravity and microgravity. Water content in **a** NP and **b** AF among Neutral 1G, Standing 0G, Neutral 0G, and Fetal Tuck 0G. NP nucleus pulposus, AF annulus fibrosus.

**Fig. 5:**
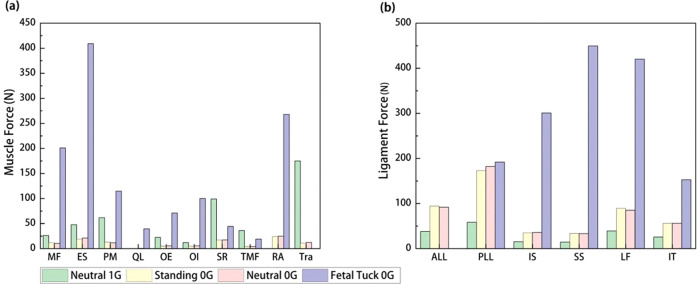
Comparison of muscle forces and ligament forces in lumbar spine among various postures in 1G gravity and microgravity. **a** Muscle forces and **b** ligament forces in lumbar spine among Neutral 1G, Standing 0G, Neutral 0G, and Fetal Tuck 0G. MF multifidus, ES erector spinae, PM psoas major, QL quadratus lumborum, OE obliquus externus, OI obliquus internus, SR semispinalis, TMF thoracic multifidus, RA rectus abdominis, Tra transversus abdominis, ALL anterior longitudinal ligament, PLL posterior longitudinal ligament, IS interspinous, SS supraspinous, FL flavum, IT intertransverse.

The mechanical responses in the lumbar spine under Neutral 1G, Standing 0G, Neutral 0G, and Fetal Tuck 0G conditions were reported (Figs. 2–5). Results between Standing 0G vs Neutral 0G were not significantly different (Figs. 2–5), thus in the following results, we focused mainly on comparing differences between Neutral 1G, Neutral 0G, and Fetal Tuck 0G since these three postures are commonly experienced by astronauts on the earth and in a microgravity environment.

### Compressive forces on lumbar discs

#### Neutral 0G vs Neutral 1G

Compared to values at Neutral 1G, the compressive forces on the lumbar discs decreased at Neutral 0G (Fig. 2a). It decreased by 70.2, 62.1, 63.5, 63.1, and 66.7% on L1L2, L2L3, L3L4, L4L5, and L5S1 discs, respectively.

#### Fetal tuck 0G vs Neutral 0G

Compared to values at Neutral 0G, compressive forces increased at fetal tuck 0G. It increased by 932.2, 785.6, 742.9, 633.2, and 577.3% on L1L2, L2L3, L3L4, L4L5, and L5S1 discs, respectively (Fig. 2a).

#### Fetal tuck 0G vs Neutral 1G

Compared to values at Neutral 1G, the compressive force was larger at fetal tuck 0G. It was 207.6, 235.9, 207.3, 170.6, and 225.3% larger on L1L2, L2L3, L3L4, L4L5, and L5S1 discs, respectively (Fig. 2a).

### Shear forces on lumbar discs

#### Neutral 0G vs Neutral 1G

Compared to values at Neutral 1G, the shear force decreased. It decreased by 79.6, 92.1, 84.4, and 47.0% on L2L3, L3L4, L4L5, and L5S1 discs, and it changed from 65 N to −16 N on the L1L2 disc at Neutral 0G, “−” means the change of force direction (Fig. 2b).

#### Fetal tuck 0G vs Neutral 0G

The shear forces at fetal tuck 0G increased by 2062.9, 643.4, and 264.5% on L1L2, L4L5, and L5S1 discs, and it changed from 20 N to −101 N and 7 N to −17 N on the L2L3 and L3L4 discs, compared to those at Neutral 0G, “−” means the change of force direction (Fig. 2b).

#### Fetal tuck 0G vs Neutral 1G

The shear force at fetal tuck 0G was 15.7 and 93.2% larger than those at Neutral 1G on L4L5 and L5S1 discs. It changed from 65 N to −354 N, 98 N to −101 N, 86 N to −17 N on the L1L2, L2L3, and L3L4 discs from Neutral 1G to fetal tuck 0G, “−” means the change of force direction (Fig. 2b).

### Disc morphology

#### Neutral 0G vs Neutral 1G

Compared to values at Neutral 1G, the disc height, cross-sectional area, and volume increased at Neutral 0G (Fig. 3). The height increased by 3.0, 2.3, 2.1, 2.1, and 2.6%, the disc cross-sectional area increased by 6.1, 4.6, 4.2, 4.3, and 5.2%, and the disc volume increased 9.3, 7.0, 6.4, 7.0, and 7.9% in L1L2, L2L3, L3L4, L4L5, and L5S1 discs, respectively (Fig. 3).

#### Fetal tuck 0G vs Neutral 0G

Compared to results at Neutral 0G, the disc height, cross-sectional area, and volume decreased at fetal tuck 0G (Fig. 3). The disc height decreased by 10.7, 9.9, 8.3, 7.5, and 7.0%, the disc cross-sectional area decreased 20.3, 18.9, 15.8, 14.4, and 13.5%, and the disc volume decreased 28.9, 26.9, 22.8, 20.9, and 19.6% in L1L2, L2L3, L3L4, L4L5, and L5S1 discs, respectively (Fig. 3).

#### Fetal tuck 0G vs Neutral 1G

Compared to results at Neutral 1G, the disc height, cross-sectional area, and disc volume were smaller at fetal tuck 0G (Fig. 3). The height was 8.0, 7.9, 6.4, 5.5, and 4.6% smaller, cross-sectional area was 15.4,15.1, 12.3,10.7, and 9.0% smaller, and the disc volume was 22.2, 21.8,17.9, 15.7, and 15.4% smaller in L1L2, L2L3, L3L4, L4L5, and L5S1 discs, respectively (Fig. 3).

### Water content

#### Neutral 0G vs Neutral 1G

Compared to results at Neutral 1G, the water content increased at Neutral 0G. It increased by 5.2, 3.9, 3.6, 3.7, and 4.4% in L1L2, L2L3, L3L4, L4L5, and L5S1 discs, respectively (Fig. 4).

#### Fetal tuck 0G vs Neutral 0G

Compared to results at Neutral 0G, the water content decreased at fetal tuck 0G, it decreased by 18.8, 17.3, 14.3, 12.9, and 12.0% in L1L2, L2L3, L3L4, L4L5, and L5S1 discs, respectively (Fig. 4).

#### Fetal tuck 0G vs Neutral 1G

Compared to results at Neutral 1G, the water content was smaller at fetal tuck 0G. It was 14.5, 14.1, 11.3, 9.7, and 8.1% smaller in L1L2, L2L3, L3L4, L4L5, and L5S1 discs, respectively (Fig. 4).

### Muscle force

#### Neutral 0G vs Neutral 1G

Compared to results at Neutral 1G, muscle forces decreased in most regions at Neutral 0G (Fig. 5a). The total force in MF, ES, PM, OE, OI, SR, TMF, and Tra groups in the lumbar regions decreased 59.8, 55.7, 81.1, 75.7, 53.3, 82.5, 88.1, and 92.9%, while the total force in QL and RA muscle groups slightly increased, with an increase of 1 N (from inactivated state) and 25 N (from inactivated state), respectively.

#### Fetal tuck 0G vs Neutral 0G

Compared to values at Neutral 0G, muscle forces increased in most regions at fetal tuck 0G. It increased by 1820.1%, 1828.8, 882.3, 7165.5, 1210.2, 1682.2, 154.1, 342.2, and 979.5% in MF, ES, PM, QL, OE, OI, SR, TMF, and RA, while the total force in Tra muscle group decreased from 12.3 N to 0 N (deactivated) (Fig. 5a).

#### Fetal tuck 0G vs Neutral 1G

The muscle forces at Fetal Tuck 0G were larger compared to those at Neutral 1G (Fig. 5a). It was 7.7, 8.5, 1.9, 3.2, and 8.3 times those at Neutral 1G in MF, ES, PM, OE, and OI muscles. In QL, it was 40 N at Fetal Tuck 0G and 0 N in Neutral 1G. In RA, it was 268 N at fetal tuck 0G and 0 N at Neutral 1G. It decreased in SR, TMF, and Tra muscles.

### Ligament force

#### Neutral 1G vs Neutral 0G

Compared to values at Neutral 1G, the ligament forces increased at Neutral 0G, increased 142.6, 211.6, 133.0, 127.0, 117.0%, and 121.1% in ALL, PLL, IS, SS, FL, and IT, respectively (Fig. 5b).

#### Fetal tuck 0G vs Neutral 0G

The ligament forces increased at fetal tuck 0G, compared to those at Neutral 0G (except ALL ligament) (Fig. 5b). It increased 5.3, 744.9, 1258.7, 394.5, and 171.8% in PLL, IS, SS, FL, and IT, respectively. It decreased from 92 N to 0 N in ALL.

#### Fetal tuck 0G vs Neutral 1G

The ligament forces at fetal tuck 0G was larger compared to those at Neutral 1G (except ALL ligaments) (Fig. 5b). It was 228.2, 1868.9, 2984.4,1945.8, and 650.0% larger in PLL, IS, SS, FL, and IT. It was 38 N smaller in ALL (i.e., 38 N in Neutral 1G, 0 N in fetal tuck 0G).

### Variation analysis

Our results showed that when the disc height varied in the range of [−20%, 20%] of the original height, our simulated disc compressive force varied in the range of [−1.3%, 0.9%] (values were averaged over five lumbar discs, same for the following data), muscle force in the range of [−4.6%, 3.3%], ligament force in the range of [4.7%, −3.4%], and disc height change in the range of [14.7%, −11.7%] of the original values, respectively. When the disc cross-sectional area varied in the range of [−20%, 20%] of the original area, our simulated disc compressive force varied in the range of [1.4%, −1.5%], muscle force in the range of [5.6%, −5.8%], ligament force in the range of [−7.6%, 6.9%], and disc height change in the range of [14.0%, −6.9%] of the original values, respectively.

## Discussion

In this study, the mechanical change of various spinal segments in the lumbar regions, including disc load, disc swelling, disc morphology, muscle force, and ligament force were quantitatively analyzed and compared among Neutral 1G, Neutral 0G, and fetal tuck 0G. Our results showed that discs compressive forces, shear forces, and muscle forces decreased significantly at Neutral 0G, while the disc water content, disc height, cross-sectional area, volume, and ligament forces increased at Neutral 0G, compared to those at Neutral 1G. The fetal tuck position at 0G showed a reverse effect on these changes seen at Neutral 0G, with values much larger than those at Neutral 1G.

Both compressive and shear forces on lumbar discs decreased in microgravity at neutral postures, causing the water to flow into discs due to unbalanced osmotic pressure in the disc and the lowered external forces on the disc, leading to the increase in water content in the discs. The increased water content caused the discs to swell, leading to larger disc size, as seen in increased disc height, area, and volume.

Our simulated disc size change was reasonable with experimental data. Early studies reported that astronaut stature increased up to 3% during flight^4,12^, recently Yong and Rajulu reported that the seated height of astronauts increased by 4 ± 1%^5^. According to Styf et al. that 35 to 60% of the spinal elongation is due to increases in intervertebral disc height^6^, thus the disc height increase in Yong and Rajulu’s study would be 1.4 ± 0.35% to 2.4 ± 0.6%. Our simulated disc height change (i.e., 3.0, 2.3, 2.1, 2.11, and 2.6% for L1L2, L2L3, L3L4, L4L5, and L5S1 discs) are close to this range. Treffel et al. found that after 3-day exposure to simulated microgravity through dry immersion, disc volume increased by 8 ± 9% (T12-L1) and 11 ± 9% (L5S1)^13^. Our simulated disc volume increase (e.g., 6.4–9.3%) were close to these experimental data.

It is proposed that expansion of the disc in microgravity may cause deformation of collagen in the annulus fibrosis, surpassing the physiological range of 3–4%, resulting in stimulation of the Type IV mechanoreceptors/free nerve endings, which might cause the sinuvertebral nerves to continually transmit impulses, eventually resulting in a perception of low back pain^14^. Our results on disc cross-sectional area and volume increases at Neutral 0G were in the range of 4.2–6.1% and 6.4–9.3%, respectively, which may lead to deformation of the collagen in the annulus fibrosus larger than the 3–4% range mentioned above, thus increasing the risk of nerve stimulation in the related area and possibly causing pain.

Our results showed that the “fetal tuck” posture in microgravity may be beneficial in counteracting those spinal changes seen in Neutral 0G. The disc compressive force, shear force, disc height and volume, and disc water content all reversed back toward the values at Neutral 1G. This may help explain biomechanically why astronauts find that the “fetal tuck” posture helps relieve back pain in microgravity. However, the magnitudes of these changes at Fetal Tuck 0G far surpassed the values at Neutral 1G. For example, the compressive loads on the lumbar discs at Fetal Tuck 0G were, on average, 2.9 times the value at Neutral 1G, reaching 936 N–1266 N. It was reported that cyclic compressive force (1 Hz) at 867 N for 24 h causes disc herniations in a porcine cervical disc, which is proposed to be closest to human lumbar spines in anatomy and biomechanical characteristics^15^. The compressive forces at fetal tuck posture in our simulation are much higher than this value, such a large load on the discs may increase the risk of disc fissure and/or disc herniation^16^.

Disc load change in microgravity may also increase the risk of disc degeneration through deregulating the synthesis of the glycosaminoglycan (GAG), one of the crucial biochemical components of the disc matrix, the loss of which causes disc degeneration^17^. Studies have shown that GAG synthesis is significantly affected by mechanical loading, with the GAG synthesis rate decreased significantly with the load deviating (either increasing or decreasing) from the optimum range^18–22^. Gao et al. showed that the GAG synthesis rate decreased by 74% at a load three times the optimal load, and decreased by 80% at a load 0.1 times the optimal load at the end of an 8-h creep in the NP^23^. Since the disc load in Neutral 0G decreased to 0.3 times that in the Neutral 1G, and in fetal tuck 0G increased to 2.9 times that in the Neutral 1G, we speculate that the GAG synthesis rate would decrease significantly in both postures at microgravity. Actually, GAG content decrease has been observed by experimental studies^24,25^. For example, Jin et al. found downregulated GAG content in simulated microgravity on the earth in mice disc^24^. Fitzgerald et al. found loss of proteoglycan in the articular cartilage (which is similar to intervertebral disc both in composition and axial weight-bearing functions) of mice exposed to microgravity for 30 days on the BION-M1 craft^25^. These decreases in GAG synthesis rate in both postures at microgravity may lead to disc degeneration^26^.

Muscle forces decreased in the neutral posture in microgravity, this may cause muscle atrophy, a widely observed phenomenon among astronauts returned from long-term microgravity exposure^27–29^. At fetal tuck 0G, the muscle forces increased significantly, much larger than those at the Neutral 1G, and the maximum muscle activation level increased significantly (in the range of 10–43%), this posture may be helpful in maintaining high muscle force thus preventing lumbar muscles from atrophy, however, it may be detrimental to other mechanical segments, such as discs.

The ligament forces increased in Neutral 0G, due to that disc swelling in microgravity stretched the ligament, resulting in increases in ligament length and force. At Fetal Tuck 0G, the ligament forces continued to increase (except ALL), due to that at ‘fetal tuck’ posture, the spine flexed forward, causing most of the ligaments to stretch even longer. While the ALL ligament shortened due to the forward bending, thus resulting in a decrease in its force. The large increase in the ligament forces at the “fetal tuck” posture may increase the risks of ligaments damages. Our calculated forces in the FL ligament at L1-L4, the IS ligament at L1-L3, and the SS ligament at L2-L4 were larger than the failure forces measured by Pintar et al.^30^ and Cornaz et al.^31^ (Table 1).

**Table 1:** Ligament forces predicted at fetal tuck 0G (fetal tuck), compared to failure forces from literature (Fail).

Parameter	Ligament	L1L2		L2L3		L3L4		L4L5		L5S1	
		Fetal tuck	Fail	Fetal Tuck	Fail	Fetal tuck	Fail	Fetal tuck	Fail	Fetal tuck	Fail
Fetal tuck 0G force(N)	ALL^a^	0.0	415.27	0.0	496.39	0.0	401.23	0.0	489.29	0.0	258.27
	PLL^a^	25.3	366.8	90.0	909.8	57.8	389.1	18.7	659.0	0.0	628.7
	IT^a^	49.2	304.2	41.2	434.5	32.2	236.5	20.0	108.0	10.1	171.8
	FL^b^	**122.8**	59	**147.9**	59	**100.2**	59	38.2	59	11.2	59
	IS^a^	**90.3**	74.8	**91.1**	40.9	68.8	87.4	45.9	84.6	4.7	122.0
	SS^a^	142.3	169.0	**128.7**	55.4	**97.9**	52.8	71.3	85.9	9.4	168.9

*ALL* anterior longitudinal ligament, *PLL* posterior longitudinal ligament, *IT* intertransverse, *FL* flavum, *IS* interspinous, *SS* supraspinous.

^a^ From Pintar et al. (1992)^39^.

^b^From Cornaz et al. (2021)^40^.

The bold values are higher than the fail values measured in literature (Column ‘Fail’), indicating the corresponding ligaments may be in higher risk of damage.

Our variational analysis results indicate that variations in disc height and the cross-sectional area may not significantly influence forces on lumbar discs, muscle forces, and ligament forces, it may influence the disc height change at fetal tuck 0G. Our results found a negative correlation between disc size and disc height change, at larger disc size (either through larger disc height or disc cross-sectional area), the disc height change was smaller compared to that at smaller disc size.

There are some limitations in this study. One limitation is that in modeling the disc’s mechanical behavior, linear relationships were used for translational and rotational behaviors, and more complex and realistic mechanical models, such as creep, were not considered in our model. This simplification may affect the deformation of the disc and forces on the muscle and ligaments. Another limitation is that in modeling muscle, the muscle strength was assumed to be cross-sectional area dependent, this simplification may affect the muscle forces and other segmental force calculations, in the future, more realistic muscle mechanical models, will be considered. In addition, even though this model was well validated in the 1G environment, it was only partially validated due to limited experimental data available in microgravity conditions, we will keep validating our model when more experimental data are available in the future. Another limitation is that the angles for fetal tuck posture used in this study was an estimation from the gesture due to the lack of data. This estimation may be different from real situations and may affect the forces calculated.

In conclusion, in this study, we quantitatively analyzed and compared the changes in intervertebral disc load, disc water content, disc morphology (height, cross-sectional area, volume), muscle forces, and ligament forces in the lumbar spine among Neutral 1G, Neutral 0G, and fetal tuck 0G conditions using a musculoskeletal modeling approach. Our results showed that lumbar discs compressive forces, shear forces, and muscle forces decreased significantly at Neutral 0G, while the disc water content, disc morphology, and ligament forces increased at Neutral 0G, compared to those at Neutral 1G. The fetal tuck 0G showed reverse effects on these changes seen at Neutral 0G, with magnitudes much larger than those at Neutral 1G, which may increase the risk of damage to discs, muscles, and/or ligaments. Our results are important for understanding the biomechanical mechanisms of microgravity-related disc health, and this study provides a tool for quantifying mechanical changes in various spinal segments under various gravitational environments.

## Methods

### Theoretical studies

The effects of microgravity on the biomechanical changes of disc load, disc swelling (water content), disc morphology (height, volume, cross-sectional area), muscle force, and ligament forces in the lumbar regions were studied using a full-body musculoskeletal model developed with the AnyBody Modeling System (AnyBody Technology, Version 7.3, Denmark). The anatomical structure and sizes of the body segments were from a male with a height of 1.74 m and a weight of 72 kg^32^. Specifically, in the lumbar spine, the model includes five lumbar vertebrae, five intervertebral discs, ten major muscle groups [including lumbar multifidus (MF), erector spinae (ES), psoas major (PM), quadratus lumborum (QL), obliquus externus (OE), obliquus internus (OI), semispinalis (SR), thoracic multifidus (TMF), rectus abdominis (RA), and transversus abdominis (Tra)], and six lumbar ligament groups [including anterior longitudinal ligament (ALL), posterior longitudinal ligament (PLL), interspinous (IS), supraspinous (SS), flavum (FL), and intertransverse (IT)].

For the mechanical behaviors, the vertebral bones were modeled as rigid bodies. The intervertebral discs were modeled as 6 degrees of freedom joints with linear momentum-rotational deformation and linear force-translational deformation relationships^33^:

1 $$F_i = k_ix_i,$$

2 $$M_i = h_i\theta _i,$$

where *F*_*i*_ is the reaction force on the disc, *x*_*i*_ is the translational displacement along the *i*^th^ axis (*i* = anterior-posterior, proximal-distal, left-right lateral direction), *M*_*j*_ is the reaction moment on the disc, *θ*_*i*_ is the rotational angle along the *i*th axis, *k*_*i*_ is the translational stiffness and *h*_*i*_ is the rotational stiffness of the disc, with values from the literature^33^. The joint rotational centers for flexion were set with fixed values taken from the literature^34^.

To simulate the swelling effects of lumbar discs during unloading in microgravity, the deformation-dependent swelling pressure of the intervertebral disc was introduced by the following equation^35^:

3 $$F_s = {\mathrm{RT}}\left( {\sqrt {c^{F^2} + 4c^{ \ast ^2}} - 2c^ \ast } \right),$$

where *R* is the universal gas constant (8.3144 JK^−1^ mol), *T* is the temperature in Kelvin (310.15 K), c^*^ is the concentration of Na^+^ and Cl^−^ in the surrounding environment of the discs (150 mM). *c*_*F*_ is the fixed charge density (FCD) inside the disc, which is dependent on disc deformation as follow^35^:

4 $$c^F = c_0^F\frac{{\phi _0^w}}{{\phi _0^w + J - 1}},$$

Where $c_0^F$ is FCD inside the disc at the reference state (i.e., neutral posture at 1G gravity, values were listed in Table 2), *J* is the volume ratio of the disc between the deformed and reference state. Assuming that during swelling, the percentage changes in disc dimension were approximately similar in all three principle directions, the volume ratio was estimated by: *J* = (*h*/*h*_0_)^3^, where *h* is disc height after deformation and *h*_*0*_ is disc height at reference state (with values listed in Table 2).

The water content in the disc is deformation dependent and is calculated as follow^35^:$\phi ^w = \frac{{\phi _0^w + J - 1}}{J}$, where *ϕ*^*w*^ is disc water content after deformation, and $\phi _0^w$ is disc water content at the reference state (with values listed in Table 2).

**Table 2:** Parameters for lumbar disc height (*h*_0_), cross-sectional area (*A*_0_), water content $\left( {\phi _0^w} \right)$, and fixed charge density $\left( {c_0^F} \right)$ at Neutral 1G condition.

	*h*_0_ [mm]	*A*_0_ [mm^2^]	$\phi _0^w$		$c_{0NP}^F$ [mol/m^3^]
			NP	AF	
L1L2	9	1425	0.85	0.775	261
L2L3	10.4	1658			242
L3L4	11.5	1714			239
L4L5	11.8	1684			215
L5S1	11.3	1709			217

The compressive load on the disc (*F*_ext_) due to body weight, muscle forces, and ligament forces (in a direction perpendicular to the lower surface of the disc) was assumed to consist of two forces, namely, a swelling force (*F*_*S*_) generated by the swelling pressure, and an elastic force (*F*_*E*_) generated by disc deformation. It was calculated as:

5 $$F_{{\mathrm{ext}}} = F_S + F_E,$$

In this study, the average FCD in annulus fibrosus (AF) was assumed to be 80% of that in the nucleus pulposus (NP) for healthy discs based on experimental data^36^, and the cross-sectional area of NP was assumed to be 40% of the whole disc cross-sectional area, also based on experimental data^37^. The swelling pressure in the lumbar discs was estimated as:

6 $$F_S = A_{{\mathrm{disc}}}{\mathrm{RT}}\left[ {0.4\left( {\sqrt {c_{{\mathrm{NP}}}^{F^2} + 4c^{ \ast ^2}} - 2c^ \ast } \right) + 0.6\left( {\sqrt {c_{{\mathrm{AF}}}^{F^2} + 4c^{ \ast ^2}} - 2c^ \ast } \right)} \right],$$

where *A*_disc_ is the disc cross-sectional area, $c_{{\mathrm{NP}}}^F$ is the mean FCD in the NP, and $c_{{\mathrm{AF}}}^F$ is the mean FCD in the AF. The average water content in the disc was estimated by:$\phi ^w = 0.4\phi _{{\mathrm{NP}}}^w + 0.6\phi _{{\mathrm{AF}}}^w$, where $\phi _{{\mathrm{NP}}}^w$ and $\phi _{{\mathrm{AF}}}^w$ are the water content in the NP and AF, respectively.

The ligaments were modeled as piecewise linear models, in which the stiffness is dependent on the strain, with values taken from experimental results by Chazal et al.^38^. The values for the stiffness can be seen in Baldoni and Gu^33^ and listed briefly in Table 3. For the muscle, the maximum muscle strength was assumed to be its functional cross-sectional area dependent, similar to that in the literature^39^. The values listed in Tables 2, 3 were the same in 1G and 0G conditions.

**Table 3:** Ligament stiffness values in the model.

Ligament	Stiffness (N/mm)	Strain (%)
Anterior longitudinal ligament (ALL)	36.2	<0,11>
	115.9	<11, 41>
	43	<41, 51>
Posterior longitudinal ligament (PLL)	52.7	<0,11>
	127	<11,28>
	37.1	<28,37>
Interspinous (IS), Supraspinous (SS)	13	<0,14>
	38.5	<14,36>
	10.3	<36,48>
Flavum (FL)	23.4	<0,8>
	54.5	<8,20>
	12.5	<20,25>
Intertransverse (IT)	12.5	<0,9>
	61.4	<9,15>
	25	<15,17>

This model has been primarily validated against experimental data^32^ under various daily postures^40^. The compressive forces simulated with this model at 1G condition were compared well with the in vivo human data^32^ at 12 different everyday postures (including lying supine, sitting slouched, sitting straight, standing, standing with 36° flexion, standing with 19° extension, standing with 24° rotation to the left, standing with 17° rotation to the right, standing with 18° bent to the right, standing with a weight lifted close to the chest, standing with a weight lifted while flexed forward, and standing with a weight lifted with arm stretched), details could be seen in our previous publication^40^. Further validation in the 0G condition was included in the discussion below.

### Numerical modeling

In this study, the neutral body posture (e.g., relaxed floating) in microgravity (denoted as “Neutral 0G”), the “fetal tuck” posture in microgravity (denoted as “Fetal Tuck 0G”), and for comparison, the neutral body posture (e.g., relaxed standing) in 1G gravity (denoted as “Neutral 1G”) and the neutral body posture (e.g., relaxed standing) in 0G gravity (denoted as “Standing 0G”) were simulated (Fig. 1). The images of the full-body musculoskeletal model shown in Fig. 1 were from the Anybody database (AMMR 2.3.4) which were originally developed by de Zee et al. ^39^, Ignasiak et al. ^41^, Maganaris^42^, Dostal and Andrews^43^, Herzog and Read^44^, and Hintermann, Nigg, and Sommer^45^.

The Neutral 1G and Standing 0G posture was simulated as follows: the sternoclavicular joint protraction was 23° and sternoclavicular joint elevation was 11.5°. The glenohumeral flexion was 8°, abduction was 10°, the elbows were flexed forward 8°, elbow pronation was −20°, the hip flexion was −6°, the hip abduction was 5°, and the rest of the joint angles were set to 0° (Fig. 1a).

The Neutral 0G was simulated according to data from NASA^10^: the neck was bent forward 24° and the line of sight was lowered 15° compared to that in Neutral 1G. The glenohumeral flexion was 39°, the abduction was 35°, the elbows were flexed forward 77°, the elbow pronation was 60°, the hip flexion was 55°, the hip abduction was 16°, the hip external rotation was 17°, the knee flexion was 55°, and the ankle plantar flexion was 21°. The rest of the joint angles were set to 0° (Fig. 1b).

The fetal tuck 0G was simulated as follows: the pelvis was flexed forward 80° relative to the thorax, the neck was bent forward 24°, the sternoclavicular joint protraction was 23°, the sternoclavicular joint elevation was 11.5°, the glenohumeral joint flexion was 80°, the glenohumeral joint external rotation was −90°, the elbow flexion was 90°, the hip flexion was 100°, the knee flexion was 150°, and the ankle plantar flexion was 21°. The rest of the joint angles were set to 0° (Fig. 1c).

In this study, the change of mechanical loadings on the lumbar intervertebral discs, disc swelling (water content), disc morphology, muscle forces, and ligament forces were quantitatively analyzed and compared among Neutral 1G, Standing 0G, Neutral 0G, and fetal tuck 0G conditions.

Most parameters were from experimental data that measured from a certain demographic group. To show how this may affect the reliability of our results, as an example, we varied the disc height and cross-sectional area in the range of [−20%, 20%] of the original values, based on experimental data^46^, and results under such conditions were compared to the original ones.

### Reporting Summary

Further information on research design is available in the Nature Portfolio Reporting Summary linked to this article.

## Supplementary information

Reporting Summary checklist

## Data Availability

The data that support the findings of this study are available from the corresponding author upon request.
